# Supplementary material for: Cytokines interferon−γ− inducible protein 10 and granulocyte−macrophage colony−stimulating factor are associated with psychiatric symptoms in opioid−dependent patients: A cross− sectional study
Source: PLoS One. 2025 Aug 7;20(8):e0324365. doi: 10.1371/journal.pone.0324365 (PMC12331062; doi:10.1371/journal.pone.0324365)
Supplement: S1 Table — Median cytokine values of healthy controls. (DOCX) [file pone.0324365.s001.docx]

**Supplementary table 1.** Median cytokines values of healthy controls (N=10)

| Cytokine (pg/ml) | LLOD | N (%) < LLOD | Median (Q1-Q3) |
| --- | --- | --- | --- |
| TNF | 1.08 | 0 (0) | 35 (20-55) |
| IFN-γ | 0.44 | 0 (0) | 112 (106-159) |
| IL-1β | 1.0 | 4 (40) | 1.51 (0.91-2.06) |
| IL-1Ra | 0.64 | 0 (0) | 118 (85-158) |
| IL-2 | 0.24 | 0 (0) | 4.96 (3.62-7.65) |
| IL-4 | 0.08 | 0 (0) | 4.17 (3.51-5.08) |
| IL-6 | 0.80 | 0 (0) | 4.47 (3.70-5.45) |
| IL-8 | 0.52 | 0 (0) | 7.93 (5.40-10.8) |
| IL-9 | 0.48 | 0 (0) | 71 (54-89) |
| IL-13 | 1.00 | 0 (0) | 5.96 (2.56-8.76) |
| IL-15 | 0.68 | 5 (50) | 0.61 (0.31-1.59) |
| IL-17α | 0.72 | 0 (0) | 78 (64-118) |
| MCP-1 | 0.44 | 0 (0) | 32 (20-44) |
| IP-10 | 3.64 | 0 (0) | 599 (524-847) |
| Eotaxin | 0.72 | 0 (0) | 126 (112-163) |
| MIP-1α | 1.8 | 3 (30) | 8.59 (3.23-17.4) |
| MIP-1β | 2.48 | 4 (40) | 20 (4.83-64) |
| RANTES^1^ | 2.84 | 0 (0) | 8371 (4455-10034) |
| GM-CSF | 1.2 | 0 (0) | 29 (22-38) |
| VEGF | 1.0 | 0 (0) | 76 (26-107) |

LLOD, lower limit of detection; TNF, tumour necrosis factor; IFN-γ, interferon-gamma; IL‑1β, interleukin-1beta; IL-1ra, interkeukin‑1 receptor antagonist; IL‑2, interleukin-2; IL‑4, interleukin-4; IL‑5, interleukin-5; IL‑6, interkeukin-6; IL‑8, interleukin-8 (C-X-C motif chemokine ligand 8; CXCL8); IL‑9, interleukin-9; IL‑13, interleukin- 13; IL-15, interleukin-15; IL-17, monocyte chemotactic protein (MCP-1/CCL2); IP-10, interferon-γ-inducible protein 10 (C-X-CL chemokine 10; CXCL10); MIP-1α, macrophage inflammatory protein-1α (CCL3); MIP-1β, macrophage inflammatory protein-1-β (CCL4); RANTES, regulated upon activation T cell expressed and secreted; GM-CSF, granulocyte macrophage colony stimulating factor; VEGF, vascular endothelial growth factor. ^1^RANTES were above the upper limit of the assay in 4 healthy controls.
