# Supplementary material for: Cytokines interferon−γ− inducible protein 10 and granulocyte−macrophage colony−stimulating factor are associated with psychiatric symptoms in opioid−dependent patients: A cross− sectional study
Source: PLoS One. 2025 Aug 7;20(8):e0324365. doi: 10.1371/journal.pone.0324365 (PMC12331062; doi:10.1371/journal.pone.0324365)
Supplement: Attachment — Submitted filename: Response to Reviewers.docx [file pone.0324365.s003.docx]

Response to Reviewers

Bodø, 25.01.25

Dear esteemed Academic editor and Reviewers,

By this response, the online submitted revised manuscript and through the online submission forms and procedures, we hope that all the editorial and revisional requirements have been met. The entire manuscript has been through an extensive language revision. In the “Revised manuscript with track changes”, additions in response to the editor’s and reviewer’s input are highlighted in yellow, and language edits are illustrated in red from the tracking.

Below are the responses to “Additional Editor Comments, and “Review comments to the Author”.

Additional editor comments:

We are grateful for your valuable input and your comments addressed below.

1. **Was HCV genotype considered**?

HCV genotype was not considered, but is of importance. It has been added as a point under “limitations” in the revised manuscript lines 253-255.

2. **What is the HIV status of the population?**

HIV-status was not investigated, and therefore unknown. This information has now been added in the revised manuscript lines 253-255.

Reviewer 1:

Thank you for your thorough and concise review of our paper. We hope that we have answered all your concerns below, and that the responses are to your satisfaction.

1. **One of the major issues with substance abuse-related studies is polydrug use. It is unclear whether the authors considered this issue while interpreting the results.**

Thank you for pointing out the lack of consideration of the impact polydrug use had on cytokine levels. The issue is addressed in the discussion of the revised manuscript lines 265-268.

2. **The type of opioids used may impact the study outcomes. Is there any information about the participants' opioid use history?**

Information pertaining to opioid use history (other than OMT-opioids) was limited to use in the last 30 days before inclusion and blood-sampling. This is illustrated in table 4 in the manuscript.

3. **The duration of OMT is an important factor to consider when interpreting the data.**

We agree that this is an important factor to consider and is now commented on in lines 257-259 in the revised manuscript.

4. **The relevance of these findings in the context of inflammation and substance use is not discussed.**

Placing our findings in the context of inflammation and substance use is of great relevance, and we have added a section in lines 237-240 in the manuscript.

5. **The conclusion stated, “The immunomodulatory qualities of opioids are diverse and probably influenced by both intrinsic and extrinsic factors, the complexity of which is yet to be fully understood,” is vague and not supported by the current data**.

We agree that the conclusion was vague and unsubstantiated by the data, and have revised and corrected it accordingly.

6. **Most of the cytokines measured are very low or near the lower limit of detection (LLOD), and the interquartile range (IQR) data are very close. Did the authors compare the data with a control group to validate the detection limits of the assay.**

We agree that that most of the cytokine values in the study participants were low and near LLOD and that the IQR data were very close. We did compare these data with a group of healthy controls (Supplementary table 1), and found the same there. The main reason for the variation in the lower limit of detection was that the lowest point of the standard curve varied slightly on different days. This is now commented in lines 169-172 including the addition of the Supplementary table in Supporting information.

Reviewer 1 and 2:

Both Reviewers have commented on the value of stratifying the study patients and comparing cytokine levels between the respective groups from the get-go, rather than the two with significant association to psychiatric symptoms in the entire study sample. While we agree with the reviewers’ statement that this would have been an interesting angle, the aim of this study was to characterize associations between cytokines and mental health symptoms in HCV patients. It was therefore beyond the scope of this report.

Reviewer 2:

We greatly appreciate your assessment, and hope that the answers below are to your satisfaction.

1. **Given the relationship between both inflammation AND drug seeking behaviour in females vs males an examination of gender as a variable is also needed**

Gender was taken into consideration, and included as an independent variable in the regression analysis.

2. **This leads to my next suggestion which is a discussion of the power of the study for each comparison made such that the reader can understand what may not be detected.**
   Secondary outcomes were exploratory, and a priori power calculations for secondary outcomes were not conducted.
3. **There also seems to be an opportunity to compare to HCV negative individuals using the data set from the authors previous works**

The use of the patient sample from the work referred to, would undoubtedly have added valuable strength to our study. However, these are two separate projects, each with individual approvals from the Regional ethics committee. Comparing data across the two projects was therefore not something we had approval to do.

Sincerely, on behalf of the group

Kristin Nygård-Odeh

Corresponding author
